# Peptidoglycan Crosslinking Relaxation Plays an Important Role in *Staphylococcus aureus* WalKR-Dependent Cell Viability

**DOI:** 10.1371/journal.pone.0017054

**Published:** 2011-02-28

**Authors:** Aurelia Delaune, Olivier Poupel, Adeline Mallet, Yves-Marie Coic, Tarek Msadek, Sarah Dubrac

**Affiliations:** 1 Institut Pasteur, Biology of Gram-Positive Pathogens, Department of Microbiology, Paris, France; 2 CNRS, URA 2172, Paris, France; 3 Institut Pasteur, Ultrastructural Microscopy Platform, Imagopole, Paris, France; 4 Institut Pasteur, Chemistry of Biomolecules, Department of Structural Biology and Chemistry, Paris, France; 5 CNRS, URA 2128, Paris, France; University of Liverpool, United Kingdom

## Abstract

The WalKR two-component system is essential for viability of *Staphylococcus aureus*, a major pathogen. We have shown that WalKR acts as the master controller of peptidoglycan metabolism, yet none of the identified regulon genes explain its requirement for cell viability. Transmission electron micrographs revealed cell wall thickening and aberrant division septa in the absence of WalKR, suggesting its requirement may be linked to its role in coordinating cell wall metabolism and cell division. We therefore tested whether uncoupling autolysin gene expression from WalKR-dependent regulation could compensate for its essential nature. Uncoupled expression of genes encoding lytic transglycosylases or amidases did not restore growth to a WalKR-depleted strain. We identified only two WalKR-regulon genes whose expression restored cell viability in the absence of WalKR: *lytM* and *ssaA*. Neither of these two genes are essential under our conditions and a Δ*lytM* Δ*ssaA* mutant does not present any growth defect. LytM is a glycyl–glycyl endopeptidase, hydrolyzing the pentaglycine interpeptide crossbridge, and SsaA belongs to the CHAP amidase family, members of which such as LysK and LytA have been shown to have D-alanyl-glycyl endopeptidase activity, cleaving between the crossbridge and the stem peptide. Taken together, our results strongly suggest that peptidoglycan crosslinking relaxation through crossbridge hydrolysis plays a crucial role in the essential requirement of the WalKR system for cell viability.

## Introduction

*Staphylococcus aureus* is the most common Gram-positive bacterium causing both nosocomial and community-acquired infections, which extend from superficial skin lesions to life-threatening deep-tissue invasive diseases such as endocarditis, osteomyelitis and pneumonia [1]. Asymptomatic carriage of this pathogen, usually within the nares, is widespread and often linked to *S. aureus* bacteremia [2], [3].

Despite its status as a major human pathogen, many of the mechanisms involved in *S. aureus* virulence and fitness remain poorly understood. Two-component systems (TCSs) are key regulatory pathways allowing bacteria to adapt their genetic expression to environmental changes. Composed of a sensor histidine kinase, generally bound to the cell membrane, and its cognate response regulator, they act to control expression of specific sets of genes in answer to defined signals. Most *S. aureus* strains have 16 TCSs, with an additional one present in MRSA [4], [5]. Although many of these systems remain to be characterized, several have been shown to play a role in virulence (AgrC/AgrA, SaeS/SaeR) [6], antibiotic resistance (VraS/VraR, GraS/GraR) [7]–[9], or adaptation to environmental changes such as oxygen pressure (SrrA/SrrB) [10]. Among the 16 TCSs common to all *S. aureus* strains, only one is essential for bacterial survival, the WalK/WalR system (aka YycG/YycF), highly conserved among low G+C % Gram-positive bacteria [11]–[13].

In previous work, we highlighted the major role the WalKR system plays in *S. aureus* peptidoglycan metabolism by controlling expression of most of the cell wall hydrolase genes [14]. Nevertheless, none of the identified WalKR-regulated genes appear to be essential, and the reason this signal transduction pathway is required for cell viability remains to be established. Cell wall metabolism is vital for bacterial fitness, playing an important role in cell shape and division, resistance to external stresses such as osmotic pressure, as well as host-pathogen interactions. While the role of cell wall plasticity in bacterial fitness is well documented, none of the *S. aureus* cell wall hydrolases have been characterized as essential for bacterial growth, presumably due to the high level of genomic redundancy in this pathogen, which produces several autolytic enzymes with similar activities (see MiST Database: http://genomics.ornl.gov/mist) [15], [16]. The WalKR system has also been extensively studied in *B. subtilis* and *S. pneumoniae*, and shown to be involved in regulation of cell wall metabolism in these bacteria [13], [17]–[19]. Furthermore, in *S. pneumoniae*, the essentiality of the WalKR system has been linked to the regulation of a single gene, *pcsB*, encoding a putative cell wall amidase [17], [18].

In this study, we tested whether overexpression of WalKR-regulated autolysin genes in a WalKR-independent manner could compensate for the essential nature of the system. Indeed, since the WalKR system controls the synthesis of most of the identified cell wall hydrolases, we reasoned that the essentiality of this system could be linked to global regulation of one type of autolytic activity rather than that of a single gene. This allowed us to show that only increased production of LytM or SsaA can restore cell viability in the absence of WalKR. Although the *lytM* and *ssaA* genes are not essential when WalKR is present, our results indicate that peptidoglycan crosslinking relaxation through crossbridge hydrolysis must play a crucial role in the essential nature of the WalKR system.

## Results

### WalKR depletion leads to cell wall thickening and a defect in cell division

We have previously shown that the WalKR system is a major regulator of cell wall degradation, and is also necessary for peptidoglycan biosynthesis and turn-over [14]. Since none of the identified WalKR regulon genes appear to be essential, this suggested that the WalKR requirement for cell viability could be specifically linked to its role in controlling cell wall metabolism. Cell wall plasticity is involved in daughter cell separation and mutant strains deleted for two of the major *Staphylococcus aureus* cell wall hydrolase genes (*atlA* and *sle1*) harbor an abnormal rough cell surface and a pronounced defect in cell separation [20], [21].

**Figure 1:**
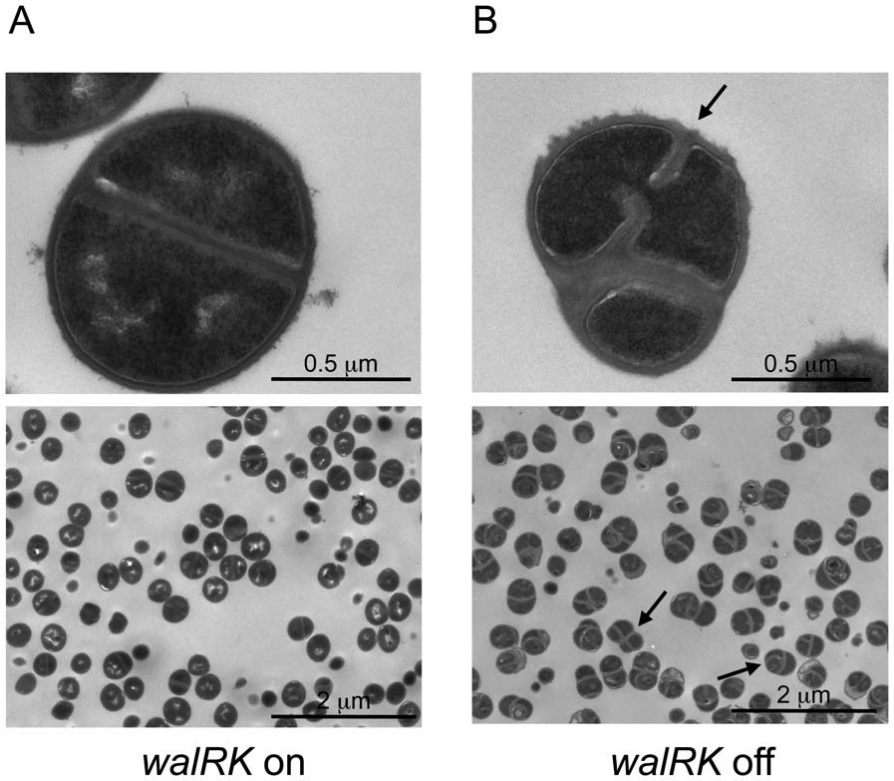
*S. aureus* starved for WalK/WalR display thickened cell walls and aberrant division septa. Strain ST1000 carrying a chromosomal P*spac-walRK* fusion was grown in TSB +/− 1 mM IPTG, harvested at OD_600nm_ = 1 (corresponding to exponential phase for the culture with IPTG and cessation of growth for the culture without IPTG) and embedded in thin sections for ultrastructure examination by transmission electron microscopy. Panel A: ST1000 grown with 1 mM IPTG. Panel B: ST1000 grown without IPTG. Bars represent 0.5 µm for the upper panels and 2 µm for the lower panels. Black arrows indicate aberrant division septa.

In order to identify morphological changes associated with WalKR-depletion, we used electron microscopy to examine *S*. *aureus* cells where the *walRK* operon is placed under the control of an IPTG-dependent inducible promoter (strain ST1000, P*spac-walRK*) [12], grown in the presence or absence of IPTG. Although no gross morphological differences were observed by scanning electron microscopy (data not shown), ultrastructural analysis by transmission electron microscopy (TEM) showed significant changes. Indeed, when WalKR is produced, the ST1000 strain displays the typical diplococcal *S*. *aureus* morphology, with a single central division septum (Fig. 1A). However, as shown in Fig. 1B, WalKR-depleted cells exhibit abnormal and misplaced division septa, with the formation of several new septa before separation of the daughter cells (indicated by arrows, Fig. 1B). WalKR-depleted cells also displayed a rougher cell surface, with amorphous “fuzzy” extracellular material, and increased cell wall thickness, almost twice that of cells producing WalKR (58.56 +/− 12.05 nm vs. 32.64 +/− 4.54 nm, respectively, Student *t* test P-value <0.05) (Fig. 1A and 1B). Interestingly, all of these phenotypes are strikingly similar to those described for *S*. *aureus atlA sle1* mutants [21] and VISA (vancomycin intermediate *S*. *aureus*) strains with reduced susceptibility to vancomycin [22].

### Uncoupling cell wall hydrolase gene expression from WalKR-dependent regulation

Given the dramatic impact of WalKR-depletion on septum placement and cell separation, we reasoned that WalKR-independent expression of one or more cell wall hydrolase genes might restore cell viability in the absence of WalKR. We therefore tested the effect of uncoupling expression of autolysin-encoding genes from WalKR-dependent regulation.

**Figure 2:**
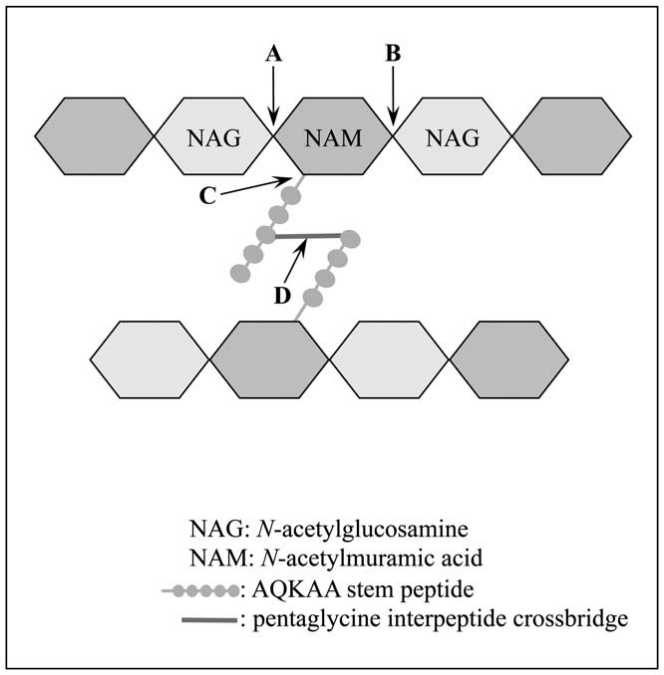
Diagram of *S. aureus* peptidoglycan and cleavage sites for WalKR-regulon encoded cell wall hydrolases. Peptidoglycan cleavage sites for WalKR-regulon encoded cell wall hydrolases are indicated: A) glycan strand glycosidic bond (glucosaminidase: AtlA). B) glycan strand glycosidic bond (lytic transglycosylases: SceD and IsaA). C) N-Acetylmuramyl-L-alanyl amide bond (AtlA amidase domain, and the Sle1, SsaA, SAOUHSC_00671, _00773, _02576 and _02883 CHAP domain hydrolases). D) pentaglycine bridge glycyl-glycyl bond (LytM endopeptidase).

**Figure 3:**
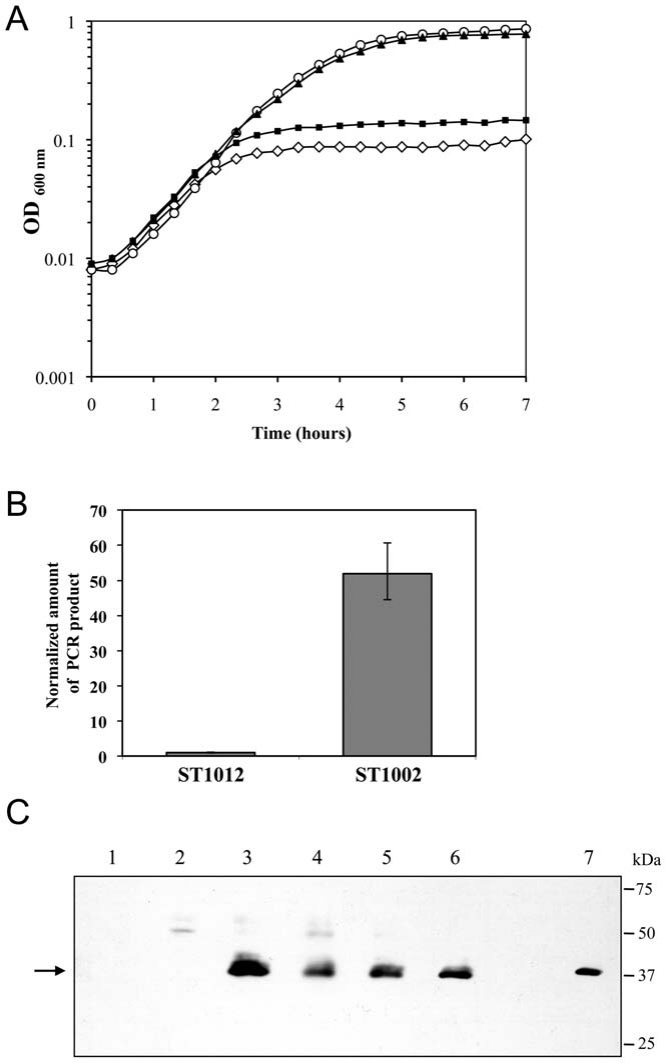
Overexpression of *lytM* restores viability to cells starved for WalKR. Cells were grown in TSB +/− 1 mM IPTG and gene overexpression was performed using the multicopy pCN51 plasmid carrying a CdCl_2_ inducible promoter whose expression was induced using 0.25 µM CdCl_2_. A) Growth curves are depicted for derivatives of *S. aureus* strain ST1000 carrying a P*spac-walRK* chromosomal fusion and the pCN51 control vector (ST1012, open symbols) in TSB-CdCl_2_ in the presence (○) or absence (⋄) of 1 mM IPTG. Growth of the ST1000 derivatives overproducing the LytM glycyl-glycyl endopeptidase (ST1002, ▴) or the inactive LytM H291A enzyme (ST1128, ▪) were carried out in TSB-CdCl_2_ without IPTG. In the presence of IPTG, strains overproducing cell wall hydrolases grew similarly to the control ST1012 strain (data not shown). B) Relative levels of *lytM* transcripts were measured by qRT-PCR during growth of strains ST1012 (pCN51 control) or ST1002 (overexpressing *lytM*), in TSB-CdCl_2_. Expression levels were normalized using the 16S rRNA as an internal standard and are indicated as n-fold change, expressed as the means and standard deviations of quadruplicate experiments. C) Western blot showing LytM and LytM H291A overproduction. Cells were grown in TSB-CdCl_2_ with or without 1 mM IPTG, and the LytM and LytM H291A proteins were detected by Western blotting using purified polyclonal rabbit antibodies. The arrow indicates the position of the mature enzyme, with a predicted molecular mass of 32 kDa. Lanes: 1, ST1012; 2, ST1012 +IPTG; 3, ST1002 (*lytM*); 4, ST1002 +IPTG; 5, ST1128 (lytM* H291A); 6, ST1128 +IPTG; 7, Purified mature his-tagged ‘LytM.

**Figure 4:**
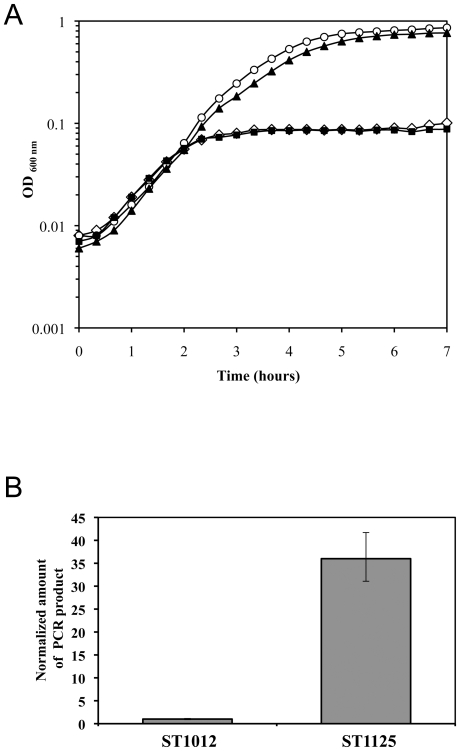
Overexpression of *ssaA* restores viability to cells starved for WalKR. Cells were grown in TSB +/− 1 mM IPTG and gene overexpression was performed using the multicopy pCN51 plasmid in the presence of 0.25 µM CdCl_2_. A) Growth curves are depicted for derivatives of *S. aureus* strain ST1000 carrying a P*spac-walRK* chromosomal fusion and the pCN51 control vector (ST1012, open symbols) in TSB-CdCl_2_ in the presence (○) or absence (⋄) of 1 mM IPTG. Growth of the ST1000 derivatives overproducing SsaA (ST1123, ▴) or inactive SsaA C171S (ST1124, ▪) was carried out in TSB-CdCl_2_ without IPTG. In the presence of IPTG, strains overproducing cell wall hydrolases grew similarly to the control ST1012 strain (data not shown). B) Relative levels of *ssaA* transcripts were measured by qRT-PCR during growth of strains ST1012 (pCN51 control) or ST1123 (overexpressing *ssaA*), in TSB-CdCl_2_. Expression levels were normalized using the 16S rRNA as an internal standard and are indicated as n-fold change, expressed as the means and standard deviations of quadruplicate experiments.

Peptidoglycan hydrolases include lytic transglycosylases cleaving the glycan strand, amidases that hydrolyze the amide bonds linking the stem peptides to the glycan strand, and glycyl-glycyl endopeptidases cleaving the *S. aureus*-specific pentaglycine interpeptide crossbridges [16]. As shown in Fig. 2, we chose to overexpress WalKR-regulated genes shown or presumed to be involved in cell wall degradation. We have recently shown that *sle1* [21], encoding a CHAP-domain amidase (Cysteine, Histidine-dependent Amidohydrolases/Peptidases) [23], [24], is a novel member of the WalKR regulon (A. Delaune *et al*., in preparation). The major autolysin gene, *atlA*, which encodes a bifunctional enzyme with amidase and glucosaminidase domains [25] was not included in this approach since we were unable to obtain an expression plasmid carrying this gene, presumably because its expression is deleterious for *E. coli*. Genes were cloned into plasmid pCN51, placing them under control of the P*cad* cadmium chloride-inducible promoter [26] (see Materials and Methods). The resulting overexpression plasmids were then introduced into strain ST1000 (P*spac-walRK*), whose growth is IPTG-dependent [12]. Growth of strain ST1000 remained IPTG-dependent in the presence of the control vector pCN51 and cadmium chloride (Fig. 3A and Fig. 4A, open symbols), indicating that neither the pCN51 vector nor the presence of cadmium chloride interfere with expression from the P*spac* promoter.

### Regulation of lytic transglycosylase genes is not responsible for WalKR essentiality

Lytic transglycosylases cleave the cell wall glycan strand. Lytic activity of IsaA and SceD has been demonstrated and we have shown that the corresponding genes are controlled by the WalKR system [14], [27]. We cloned the *isaA* and *sceD* genes in the pCN51 vector, placing them under the control of the P*cad* promoter. The resulting plasmids, pAD13 and pAD01, were introduced into strain ST1000 and IPTG-dependent growth was examined in the presence of CdCl_2_. WalKR-independent overexpression of *isaA* or *sceD* was verified by qRT-PCR, and did not allow growth of the P*spac-walRK* strain in the absence of IPTG (data not shown).

### Production of the LytM glycyl-glycyl endopeptidase restores growth to WalKR-depleted cells

The *S. aureus* NCTC 8325 core genome encodes three M23 peptidase domain proteins with putative glycyl-glycyl endopeptidase activity. Among them is the previously characterized LytM enzyme [28], [29], whereas the other two, SAOUHSC_00174 and _02464, have not been studied. Transcription of *lytM* is under the control of the WalKR system, and we have shown that WalR binds directly to its target operator sequence upstream from the *lytM* promoter [12], [14]. The SAOUHSC_00174 and _02464 genes are not preceded by a WalR binding site, and we have shown that their expression is not controlled by the WalKR system (data not shown).

As shown in Fig. 3A, a P*spac-walRK* strain overexpressing *lytM* (strain ST1002 carrying plasmid pSD3-13) grew as well in the absence of IPTG, i.e. without expression of *walRK*, as the control strain (ST1000/pCN51) in the presence of IPTG, indicating that *lytM* overexpression is able to fully restore growth and viability to cells starved for the WalKR system. We verified by quantitative real-time PCR (qRT-PCR) analysis that *walKR* expression remained inducible by IPTG, and unexpressed in the absence of IPTG in the ST1012 (ST1000/pCN51) and ST1002 (ST1000/pSD3-13) strains grown in the presence of CdCl_2_ (data not shown).

Overexpression of *lytM* and corresponding LytM overproduction were verified by qRT-PCR and Western blot analysis. As shown in Fig. 3B, pSD3-13 increases *lytM* transcription approximately 50-fold, and this increased transcription is directly correlated to significant LytM protein accumulation (Fig. 3C, lane 1 compared to lane 3). We note that *in vivo* levels of LytM protein are undetectable in the ST1000/pCN51 strain with or without WalKR production (Fig. 3C, lanes 1 and 2), although basal levels of the protein could be seen after overexposing the Western blot (data not shown). Although the genes encoding the other two M23 peptidase proteins (SAOUHSC_00174 and _02464) are not regulated by the WalKR system, we also tested their ability to compensate WalKR depletion, and showed that neither of the two proteins allowed growth of WalKR-depleted cells when overproduced (data not shown).

In order to determine whether the glycyl-glycyl endopeptidase enzymatic activity of LytM was specifically required for its capacity to restore viability to WalKR-depleted cells, we used site-directed mutagenesis to inactivate its catalytic site. The M23 peptidase domain of LytM extends from residues 208 to 309, and it has previously been shown that histidine residue 291 is crucial for LytM activity [30]. We placed the lytM* mutant allele (H291A) under control of P*cad* in pCN51, generating plasmid pSD3-24. As shown in Fig. 3A, expression of lytM* cannot restore growth to cells lacking WalKR. In order to compare LytM and LytM H291A production *in vivo* and to rule out the possibility that the inactive LytM H291A protein might be specifically targeted by proteases, we performed Western blot experiments with polyclonal LytM antibodies. As mentioned above, native levels of LytM are undetectable in the ST1000/pCN51 strain, however as shown in Fig. 3C introduction of pSD3-13 or pSD3-24 into strain ST1000 clearly led to significant overproduction of LytM (lanes 3 & 4) or LytM H291A (lanes 5 & 6), respectively.

LytM belongs to the lysostaphin-type peptidase family, whose prototype is the *S. simulans*-produced lysostaphin, which also acts as a glycyl-glycyl endopeptidase, cleaving the pentaglycine crossbridge [31]. Since active recombinant lysostaphin is readily available [32], we tested the effect of adding sub-lethal concentrations (0.01 and 0.025 µg/ml; higher amounts lead to cell lysis) to the extracellular medium on growth of the ST1000 IPTG-dependent P*spac-walRK* strain. There was no effect on the growth profile of the WalKR-depleted culture, indicating that extracellular glycyl-glycyl activity of lysostaphin cannot compensate for the absence of WalKR (data not shown).

We also tested the effect of adding purified LytM extracellularly, by purifying a His-tagged recombinant form of mature LytM, which was also unable to restore growth in the absence of WalKR, suggesting localization of the enzyme may be important (data not shown). In a reciprocal experiment, we cloned a DNA fragment encoding a recombinant active and secreted form of lysostaphin (*lss7* allele, partially deleted in the propeptide encoding region) [33] under the control of the CdCl_2_-inducible promoter of pCN51, generating the lysostaphin production plasmid pAD08. The induction conditions used (0.25 µM CdCl_2_) allowed normal growth of strain ST1000/pAD08 in the presence of IPTG. However, unlike LytM, intracellular production of lysostaphin at sub-lethal levels under conditions of WalKR depletion did not restore cell growth, suggesting that LytM and lysostaphin may differ with respect to their role in cell wall metabolism (data not shown).

### *ssaA* is the only WalKR-dependent CHAP domain amidase gene able to restore growth to a WalKR-depleted strain

CHAP domains are conserved in several peptidoglycan hydrolases, often associated with amidase activity [23], [24], [34], [35]. Careful examination of the *S. aureus* NCTC 8325 genome [5] allowed us to predict 14 genes encoding CHAP domain proteins (SAOUHSC_00256, _00427 or *sle1*, _00671, _00773, _01219, _01515, _02019, _02023, _02173, _02571 or *ssaA*, _02576, _02855, _02883, _02979). In agreement with the presence of our defined consensus WalR binding site DNA sequence, we have shown that six of these are controlled by the WalKR system: *ssaA*, SAOUHSC_00671, _00773, _02576, _02883 [14] and *sle1* (A. Delaune *et al*., in preparation).

We tested the effect of overexpressing each of these six genes on growth of the ST1000 (P*spac-walRK*) strain in the presence or absence of IPTG. Whereas WalKR-independent overexpression of *sle1*, SAOUHSC_00671, _00773, _02576 or _02883, as verified by qRT-PCR, had no effect on IPTG-dependent growth (data not shown), we showed that expression of *ssaA* from the P*cad* promoter (plasmid pAD12) fully restores growth of *S*. *aureus* in the absence of WalKR (Fig. 4A). While this work was in progress, a similar finding was independently observed by the group of Simon Foster (S. Foster, University of Sheffield, personal communication).

Growth of the ST1000 strain, starved for the WalKR system but overexpressing *ssaA*, was identical to that of the strain expressing *walRK*, and we measured *ssaA* expression from the P*cad* promoter and showed that this overexpression does not interfere with regulated expression of *walRK* from the P*spac* promoter which remained unexpressed in the absence of IPTG (Fig. 4B and data not shown respectively). As shown in Fig. 4B, *ssaA* transcription was increased approximately 30-fold in strain ST1123, carrying the pAD12 plasmid with the P*cad-ssaA* fusion, as compared to the strain carrying the control pCN51 vector under the same culture conditions (TSB with 0.25 µM CdCl_2_), similar to the *lytM* overexpression levels shown in Fig. 3B.

CHAP domains are characterized by two highly conserved motifs containing cysteine, histidine and asparagine residues, all of which have been shown to be critical for enzymatic activity [35], [36]. In order to verify whether the ability of SsaA to restore viability to WalKR-starved cells was linked to its enzymatic activity, we used site-directed mutagenesis to change cysteine residue 171 to serine, a mutation that has previously been shown to abolish the activity of PlyC, a bacteriophage lysin with a CHAP domain [37]. The mutated ssaA* (C171S) allele was placed under the control of the P*cad* promoter and introduced into strain ST1000. As shown in Fig. 4A, overexpression of the ssaA* allele is not able to restore growth to a WalKR-depleted strain. We verified by qRT-PCR that the mutated ssaA* allele was overexpressed from the Pcad promoter at levels comparable to those of the native *ssaA* allele (data not shown). Taken together, these data indicate that SsaA is the only CHAP domain protein encoded by WalKR regulon genes able to restore growth to WalKR-depleted cells and that this is dependent on its enzymatic activity.

### SsaA and LytM are not sufficient to completely compensate for the absence of WalKR

**Figure 5:**
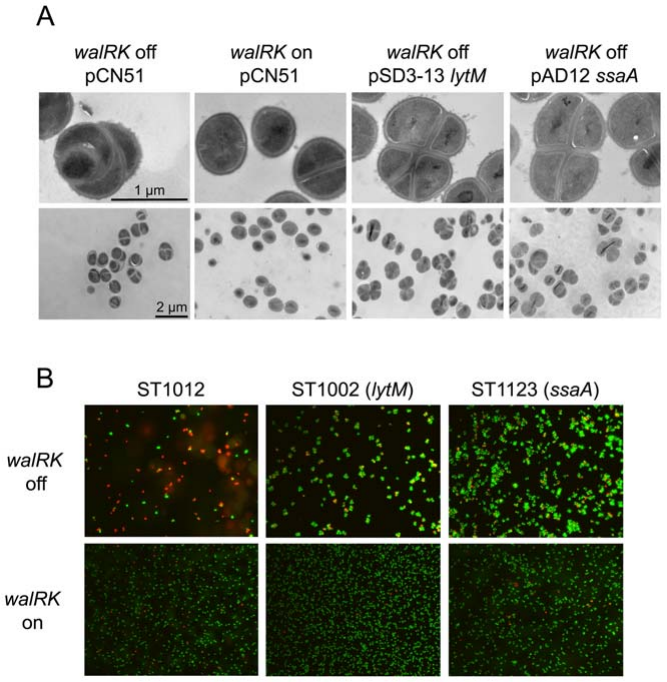
Overexpression of *lytM* or *ssaA* restores cell viability but not morphology to cells lacking the WalK/WalR TCS. Bacteria were grown in TSB CdCl_2_ 0.25 µM. To produce the WalKR system in derivatives of *S. aureus* strain ST1000 carrying a P*spac-walRK* chromosomal fusion and the indicated plasmids, 1 mM IPTG was added to the culture (*walRK* on). In the absence of IPTG, cells were depleted for WalKR (*walRK* off). A) Transmission electron microscopy ultrastructure of strains overproducing SsaA or LytM. Cells were grown in TSB-CdCl_2_ +/− 1 mM IPTG, harvested at an OD_600nm_ = 1 (corresponding to exponential phase for the culture with IPTG and cessation of growth for the culture without IPTG) and embedded in thin sections for ultrastructure examination by transmission electron microscopy. Bars represent 1 µm for the upper panels and 2 µm for the lower panels. B) Viability assay of strains overproducing SsaA or LytM using the LIVE/DEAD BacLight^TM^ assay. Two hours after reaching stationary phase, 10 ml of culture were sampled for fluorescent staining. For the control ST1012 strain without IPTG, the sample was taken 4 hours after cessation of growth following WalKR-depletion. Live bacteria appear green, following SYTO-9 staining, and dead cells are stained in red due to penetration of propidium iodide through compromised membranes.

As shown above, WalKR-depleted cells exhibit abnormal division septa and a rougher cell surface. We therefore examined the morphology of cells overproducing LytM or SsaA in the absence of WalKR, comparing strains ST1012 (pCN51), ST1002 (pSD3-13) and ST1123 (pAD12) by TEM. As shown in Fig. 5A, overexpression of *lytM* or *ssaA* only partially restored cell morphology: although cell wall thickness was diminished, cells presented a separation defect and some abnormal and misplaced division septa remained present in WalKR-depleted LytM- or SsaA-overproducing strains even though growth was indistinguishable from that of cells producing WalKR. In order to test the viability of these abnormal cells, we used fluorescence microscopy and the Live/Dead BacLight™ bacterial viability assay on stationary phase cultures of the ST1000 strain carrying either the pCN51 control plasmid or the pSD3-13 and pAD12 plasmids allowing overexpression of *lytM* or *ssaA*, respectively. As expected, viability of strain ST1000 carrying the pCN51 vector remains strictly dependent on WalKR, and almost all of the cells died when starved for WalKR (red staining, Fig. 5B upper panel). As shown in Fig. 5B, the ST1000/pSD3-13 or pAD12 strains were still viable in the absence of WalKR (green stain) but formed aggregates characteristic of bacteria with a cell separation defect (upper panel). This defect is not linked to *lytM* or *ssaA* overexpression since when the WalKR system was produced, the cells were well separated (lower panel).

## Discussion

We report here the first data establishing a direct link between WalKR-dependent regulation of cell wall metabolism and WalKR essentiality in *S. aureus*. The strategy of uncoupling expression of genes encoding cell wall hydrolases from WalKR-dependent activation allowed us to show that only two WalKR-regulon autolysin genes are able to compensate for the absence of this essential TCS, *lytM* and *ssaA*. Our results suggest that loss of cell viability following WalKR depletion can be compensated through peptidoglycan crosslinking relaxation.

LytM is produced with a signal peptide, suggesting that it is secreted, and carries a peptidase M23 domain similar to that of *S*. *simulans* lysostaphin [28]. LytM is a glycyl-glycyl endopeptidase, cleaving the pentaglycine interpeptide crossbridges of the *S. aureus* cell wall [29], [30], [38]. Cross-bridges stabilize the *S. aureus* cell wall and are essential since mutants impaired in their biosynthesis (*fmhB*, *femA, femB*) are non- or barely viable [39]–[41]. Furthermore, the pentaglycine interpeptide bridges are involved in exposure of cell surface proteins that are covalently anchored to them by sortase-dependent cross-linking [42]. Several studies have established a link between the degree of crosslinking of peptidoglycan and methicillin resistance in a PBP2′-independent manner, as well as resistance to other unrelated classes of antibiotics such as glycopeptides [39], . Pentaglycine cross-bridges thus play key roles in bacterial fitness, antibiotic resistance and in virulence, through the control of surface protein display, so it is not surprising that their biosynthesis genes are essential.

SsaA carries a carboxy-terminal CHAP domain, and its ortholog in *S. epidermidis* is highly antigenic and was recovered from whole cells and culture supernatant, suggesting it is also secreted [44]. SsaA has been linked to the hypersensitivity to macrolide-lincosamide-streptogramin B antibiotics of a thermosensitive *walRK* mutant in *S. aureus*, though the underlying molecular mechanism is unknown [45]. CHAP domains are conserved in a subset of peptidoglycan hydrolases [23], [24], [34], [35] and we have identified 14 genes encoding CHAP-domain proteins in the *S. aureus* NCTC 8325 genome [5], and shown that six of these are regulated by the WalKR system [14] and (A. Delaune *et al*., in preparation). Of these, only *ssaA* is able to compensate for the absence of WalKR when it is overexpressed (Fig. 4). Among the bacterial CHAP proteins of *S. aureus*, only Sle1, which acts as a N-acetylmuramyl-L-alanyl amidase, has been characterized [21].

Degradation of peptidoglycan is critical, since cell wall plasticity is required for cell shape and cellular division. AtlA, one of the major *S. aureus* autolysins, is a bifunctional enzyme with amidase and glucosaminidase domains, and localizes in rings associated with division septa, consistent with its requirement for daughter cell separation by splitting the crosswall [46]-[48]. The Sle1 amidase also appears to play a crucial role in splitting the septum during cell division, since a *sle1* mutant displays a higher number of septa per cell but without subsequent cell separation [21]. Interestingly, strains with mutations inactivating either *atlA* or *sle1* are viable and their growth rates are normal, with only an *atlA sle1* mutant presenting impaired growth [21].

We have shown here that WalKR depletion leads to a rougher cell surface, abnormal division septa and a defect in cell separation, but that cell viability is restored when LytM or SsaA are overproduced. Little is known about the role of these two autolytic enzymes in bacterial fitness. Unlike AtlA, LytM is not localized around the division septa, but distributed over the cell surface, suggesting that its role could be more general, involved in cell wall plasticity rather than cell division [29]. SsaA localization has never been studied. A Δ*lytM* mutant had not been described in the literature, however we constructed a mutant strain in which the entire *lytM* coding sequence was deleted and have shown that this mutant strain has no growth defect in rich medium, and behaves as the parental strain with respect to lysostaphin sensitivity and resistance to high osmolarity (data not shown). Essentiality of *ssaA* was initially controversial since although a mutant strain had been generated some time ago [45] a recent report on a genome-wide screening for essential genes in *S. aureus* using transposon-mediated differential hybridization suggested that it might be essential [49]. We constructed a Δ*ssaA* mutant as well as a Δ*lytM* Δ*ssaA* mutant (Strains ST1158 & ST1164; Table 1), and both grew as well as the parental strain, and did not display increased sensitivity to Triton X-100-induced lysis (data not shown).

**Table 1:** Strains and plasmids used in this study.

Strain or plasmid	Description	Source or reference*
**Strains**		
RN4220	Restriction-deficient transformation recipient strain	[68]
ST1000	RN4220 P*spac-walRK*	[12]
ST1002	P*spac-walRK* pSD3-13	pSD3-13ST1000
ST1012	P*spac-walRK* pCN51	pCN51ST1000
ST1020	P*spac-walRK* pAD01	pAD01ST1000
ST1031	Δ*lytM*	pAD07RN4220
ST1057	P*spac-walRK* pAD04	pAD01ST1000
ST1069	P*spac-walRK* pAD06	pAD01ST1000
ST1081	P*spac-walRK* pAD08	pAD01ST1000
ST1083	P*spac-walRK* pAD09	pAD01ST1000
ST1123	P*spac-walRK* pAD12	pAD01ST1000
ST1124	P*spac-walRK* pSD3-25	pSD3-25ST1000
ST1128	P*spac-walRK* pSD3-24	pSD3-24ST1000
ST1133	P*spac-walRK* pAD13	pAD01ST1000
ST1134	P*spac-walRK* pAD14	pAD01ST1000
ST1135	P*spac-walRK* pAD15	pAD01ST1000
ST1158	Δ*ssaA*	pAD18RN4220
ST1164	Δ*lytM* Δ*ssaA*	pAD18ST1031
**Plasmids**		
pCN51	Vector for CdCl_2_-dependent gene expression	[26]
pET28/16	Vector for protein overproduction in *E. coli*	[69]
pMAD	Allelic exchange vector	[63]
pCXlss7	Plasmid allowing lysostaphin production	[33]
pSD3-13	pCN51-*lytM*	This study
pSD3-24	pCN51-*lytM* *	This study
pSD3-25	pCN51-*ssaA* *	This study
pAD01	pCN51-*sceD*	This study
pAD04	pCN51-*sle1*	This study
pAD05	pET28/16-‘*lytM*	This study
pAD06	pCN51-*SAOUHSC_00773*	This study
pAD07	pMADΔ*lytM*	This study
pAD08	pCN51-*lss7*	This study
pAD09	pCN51-*SAOUHSC_02883*	This study
pAD12	pCN51-*ssaA*	This study
pAD13	pCN51-*isaA*	This study
pAD14	pCN51-*SAOUHSC_00671*	This study
pAD15	pCN51-*SAOUHSC_02576*	This study
pAD18	pMADΔ*ssaA*	This study

* Arrows indicate plasmid introduction by electroporation.

Although LytM is the only glycyl-glycyl endopeptidase characterized so far in *S. aureus* [28], [29], genome scanning allowed us to predict two more chromosomal genes encoding potential glycyl-glycyl endopeptidases as they share the LytM Pfam M23 peptidase domain (SAOUHSC_00174, and _02464). This likely functional redundancy could explain why the Δ*lytM* mutant has no obvious phenotype. The SAOUHSC_00174 and _02464 genes do not have a WalR binding site in their upstream region, their expression does not appear to be controlled by WalKR and their overexpression did not restore growth to cells depleted for WalKR (data not shown). The two genes appear to be expressed at a very low basal level and their roles in cell wall metabolism, if any, remains to be established.

Another well-known glycyl-glycyl endopeptidase cleaving the pentaglycine interpeptide crossbridge is lysostaphin, produced by *S*. *simulans* [50]. LytM and lysostaphin are described as having similar enzymatic activities, but we have shown that lysostaphin, added extracellularly or produced within the cells, was not able to restore viability to WalKR-depleted bacteria. This could suggest that location/targeting of LytM may be crucial or that LytM enzymatic activity could have a mild effect, allowing the cell wall to gain enough plasticity to restore growth, whereas the effect of lysostaphin has been shown to be drastic as it leads to cell lysis by forming holes in the cell wall [51]. Since distribution over the cell surface has been observed for LytM, it seems unlikely that localization is crucial for its activity [29]. The main enzymatic activities of LytM and lysostaphin are the same, i.e. glycyl-glycyl endopeptidase, but while they share a catalytic domain, they also contain other domains as well, in particular the amino-terminal half of LytM (residues 26 to 212) whose specific role remains to be established. We and others have shown that LytM is not efficient in lysing *S. aureus* cells (data not shown) while it can cleave pentaglycine bridges and modify cell wall thickness without disrupting it [28], [30], [38].

As we have shown here, overexpression of either *lytM* or *ssaA* restored cell viability, but only partially compensated cell morphology in the absence of WalKR, with a cell separation defect and abnormal, incomplete or misplaced division septa still present (Fig. 5). This partial restoration is very likely due to the fact that WalKR controls expression of both *atlA* [14] and *sle1* (A. Delaune *et al*., in preparation), since their expression is lowered in cells starved for WalKR and these remaining phenotypes are highly similar to those of a *sle1 atlA* mutant [21]. The initial finding that both LytM and SsaA can compensate for the absence of WalKR when they are overproduced was puzzling at first, since their enzymatic activities were thought to be distinct. Indeed, SsaA, with a CHAP domain, was annotated as an amidase, cleaving the N-acetylmuramoyl-L-alanyl amide bond, whereas LytM is a glycyl-glycyl endopeptidase. However, CHAP domains are in fact associated with two different types of peptidoglycan cleavage activities: N-acetylmuramoyl-L-alanyl amidase as well as D-alanyl-glycyl endopeptidase activity [23], [24], [34], [35]. This latter activity, effectively cleaving between the pentaglycine crossbridge and the stem peptide, is formally equivalent to that of LytM, since the end result of hydrolysing either the D-alanyl-glycyl or glycyl-glycyl bonds will lead to peptidoglycan crosslinking relaxation and release of a polyglycine extremity. Whereas bacterial CHAP domains are usually located at the carboxy-terminus, many *S. aureus* bacteriophage-encoded endolysins also have CHAP domains but at the amino-terminus [35]. Two of these endolysins have been well characterized: Ø11 LytA, and phage K LysK, sharing a tripartite organization: an amino-terminal CHAP domain, a central amidase2 domain, and a SH3b cell wall-binding domain. Both LytA and LysK have two distinct demonstrated enzymatic activies: a N-acetylmuramyl-L-alanyl amidase activity conferred by the amidase2 central domain and a D-alanyl-glycyl endopeptidase activity involving the CHAP domain [52], [53]. Our results, showing that SsaA behaves differently from the other WalKR-regulon encoded CHAP domain proteins, strongly suggest that the SsaA CHAP domain is also endowed with D-alanyl-glycyl endopeptidase activity, which could explain why it can act as well as LytM in restoring cell viability in the absence of WalKR.

The only other bacterium in which essentiality of the WalKR system can be bypassed by expression of a regulon gene is *Streptococcus pneumoniae*. In Streptococci, WalKR essentiality is slightly different than in *S. aureus* or *B. subtilis* since only the gene encoding the response regulator is essential [13], [54], [55]. Transcriptome analyses have shown that WalKR regulates expression of several genes involved in cell wall degradation [17]. Among them is *pcsB*, which encodes a CHAP domain protein, thought to have amidase activity, and involved in cell septation during division [17]. Interestingly, the group of Malcolm Winkler (Indiana University) was able to show that constitutive expression of the essential *pcsB* gene is sufficient to bypass the requirement of WalR for cell viability in *S*. *pneumoniae* [17]. In agreement with our results, WalKR essentiality in *S. pneumoniae* as in *S*. *aureus* appears linked to regulation of a gene involved in cell wall hydrolysis, and in both cases a gene encoding a CHAP domain protein (PcsB or SsaA, respectively) is involved.

The essential nature of the WalKR system has made it a highly attractive target for novel antimicrobial compounds [56], [57] and recent results indicate that this TCS is specifically activated in *S*. *aureus* during nasal colonization [58], [59], suggesting it may play an important role in host-pathogen adaptation and virulence and emphasizing the importance of understanding its requirement for cell viability.

We have shown here that the genes encoding LytM and SsaA are not essential in *S. aureus*, but that peptidoglycan crosslinking relaxation through pentaglycine cross-bridge cleavage is important in restoring cell viability in the absence of WalKR. Cell wall degradation is an essential process, with cross wall splitting required during cell division and peptidoglycan expansion requiring the release of free extremities to add new cell wall monomers through the activity of penicillin binding proteins. As shown in this paper, overproduction of either LytM or SsaA restores the viability of WalKR-depleted cells but not normal cell separation. We can speculate that the enzymatic activities of SsaA and LytM allow normal cell wall enlargement by increasing free polyglycine extremities, allowing cell wall biosynthesis through transpeptidase reactions. Future work will aim at understanding why this is so important in a context in which most of the cell wall hydrolases are down-regulated.

## Materials and Methods

### Bacterial strains and growth media

*Escherichia coli* K12 strain DH5α™ [F^−^ (ϕ80d*lac*ZΔM15) Δ (*lac*ZYA-*arg*F) U169 *rec*A1 *end*A1 *hsd*R17 (r_k_−, m_k_+) *pho*A *sup*E44 λ^−^ *thi*-1 *gyr*A96 *rel*A1] (Invitrogen) was used for cloning experiments, and *E*. *coli* strain BL21 λ DE3 [60] (Novagen) for protein overproduction and purification. *E. coli* strains were grown in LB medium and ampicillin (100 µg/ml) was added when required. *Staphylococcus aureus* strains and plasmids used in this study are listed in Table 1. *S*. *aureus* strain ST1000 (RN4220 P*spac-walRK*) [12] and derivatives described in Table 1 were grown in Trypticase Soy Broth (TSB; Difco) supplemented with chloramphenicol (10 µg ml^−1^), erythromycin (1 µg ml^−1^) and IPTG (1 mM) when required. Cadmium chloride (CdCl_2_) was added at a final concentration of 0.25 µM for expression from the P*cad* promoter. Bacterial growth experiments were carried out in microtiter plates (100 µl culture volume) and incubated in a Synergy 2 thermoregulated spectrophotometer plate reader (BioTek) using the Gen5^TM^ Microplate Software (BioTek). *E*. *coli* and *S*. *aureus* strains were transformed by electroporation using standard protocols [61] and transformants were selected on LB or Trypticase Soy Agar (TSA; Difco) plates, respectively, with the appropriate antibiotics.

### DNA manipulations

Oligonucleotides used in this study were synthesized by Sigma-Proligo and their sequences are listed in Table S1. *S*. *aureus* chromosomal DNA was isolated using the MasterPure^TM^ Gram-positive DNA purification Kit (Epicentre Biotechnologies). Plasmid DNA was isolated using a QIAprep Spin Miniprep kit (Qiagen) and PCR fragments were purified using the Qiaquick PCR purification kit (Qiagen). T4 DNA ligase and restriction enzymes (New England Biolabs), PCR reagents and *Pwo* thermostable DNA polymerase (Roche) were used according to the manufacturer's recommendations. Nucleotide sequencing of plasmid constructs was carried out by Beckman Coulter Genomics.

### Plasmid and mutant construction

Plasmid pCN51 carrying the P*cad-cadC* promoter module (cadmium chloride-inducible promoter and the CadC repressor gene), was used for gene expression in *S. aureus* [26]. Plasmid pET28/16, a derivative of plasmid pET28a (Novagen), was used for protein overexpression and purification [62]. The pMAD allelic replacement vector [63] was used to generate the Δ*lytM* and Δ*ssaA* mutant strains.

To uncouple gene expression from WalKR-dependent activation, DNA fragments corresponding to the coding sequences of cell wall hydrolase genes with their associated ribosome-binding sites were generated by PCR using *S*. *aureus* RN4220 chromosomal DNA and the oligonucleotide pairs listed in Table S1, introducing *Bam*HI and *Eco*RI sites at the 5′ and 3′ ends of the DNA fragments, respectively. The lytM* (encoding the LytM H291A protein) and ssaA* (encoding the SsaA C171S protein) mutant alleles were generated by site-directed mutagenesis using strand-overlap extension PCRs (SOE-PCR) [64]. Briefly, two DNA fragments overlapping at their ends were synthesized by PCR using oligonucleotide pairs OSA215/OSA259 and OSA260/OSA217 for lytM* and OAD042/OSA265 and OSA266/OAD043 for ssaA* and fused by SOE-PCR using the external oligonucleotides, (OSA215/OSA217 for lytM*, OAD042/OAD043 for ssaA*) introducing *Bam*HI and *Eco*RI sites at the 5′ and 3′ ends of the DNA fragments. The DNA fragments were cloned between the corresponding restriction sites of plasmid pCN51 in order to place gene transcription under the control of the P*cad* promoter. The resulting plasmids are listed in Table 1. DNA ligations were transformed into *E. coli* DH5α™ and the nucleotide sequences of the DNA inserts were determined before introducing the plasmids into the *S. aureus* ST1000 strain.

For intracellular production of lysostaphin in *S*. *aureus*, a 897-bp DNA fragment encoding an active recombinant form of lysostaphin (*lss7*) was generated by PCR using plasmid pCXlss7 [33], and oligonucleotides OP274 and OSA252 (see Table S1), and cloned between the *Bam*HI and *Eco*RI sites of the pCN51 vector, resulting in plasmid pAD08.

Mature LytM (without its signal peptide, residues 26 to 316) was overproduced in *E*. *coli* using plasmid pAD05, constructed by cloning a 887-bp PCR-generated *Nco*I/*Xho*I DNA fragment corresponding to ‘*lytM* (oligonucleotide pair OAD027/OAD018) between the corresponding restriction sites of plasmid pET28/16, replacing the stop codon with an *Xho*I restriction site. This allows the creation of a translational fusion adding six histidine residues to the carboxy-terminus of the corresponding proteins, placing expression of the genes under the control of a T7 bacteriophage promoter.

For generation of a Δ*lytM* mutant strain, two DNA fragments, of 615 and 804 bp, were generated by PCR using oligonucleotides OAD003/OAD004 and OAD013/OAD014, respectively (see Table S1), corresponding to the DNA regions located immediately upstream and downstream from the *lytM* gene. These DNA fragments were cloned in tandem in two consecutive steps, between the *BamH*I and *Bgl*II restriction sites of the pMAD vector, resulting in plasmid pAD07. The plasmid was introduced by electroporation into *S*. *aureus* strain RN4220, and transformants were selected at 30°C on TSA plates containing erythromycin and 5-bromo-4-chloro-3-indolyl-β-D-galactopyranoside (X-Gal) (100 µg/ml). Integration and excision of pAD07 were then performed as previously described [63], yielding strain ST1031 (Δ*lytM* mutant).

The same approach was used to obtain the Δ*ssaA* mutant. Oligonucleotides OAD064/OAD065 and OAD066/OAD067 were used to generate by PCR two DNA fragments of 559 bp and 607 bp respectively (Table S1). These fragments, corresponding to the upstream and downstream regions of the *ssaA* gene, were cloned between the *BamHI* and *NcoI* restrictions sites of the pMAD vector to construct pAD18. The plasmid was then introduced into *S*. *aureus* strain RN4220 or ST1031 (Δ*lytM*). Integration and excision of pAD18 were performed as previously described [63], yielding strains ST1158 (RN4220 Δ*ssaA*) and ST1164 (Δ*lytM* Δ*ssaA*). The markerless gene deletions were confirmed by PCR amplification.

### Total RNA extraction

Strains were grown in TSB- CdCl2 (0.25 µM) +/− 1 mM IPTG at 37°C, with aeration until OD_600_ = 1. Cells were pelleted by centrifugation (2 min, 20,800×g) and immediately frozen at −20°C. RNA extractions were then performed as previously described [65], followed by a DNase I treatment with the TURBO DNA-free reagent (Ambion, Austin, TX) in order to eliminate residual contaminating genomic DNA.

### cDNA synthesis and quantitative real time PCRs (qRT-PCR)

cDNA synthesis was carried out as previously described [14]. Oligonucleotides were designed with the BEACON Designer 4.02 software (Premier Biosoft International, Palo Alto, CA) in order to synthesize 100–200 bp amplicons (see Table S1). Quantitative real-time PCRs (qRT-PCRs), critical threshold cycles (CT) and *n*-fold changes in transcript levels were performed and determined as previously described using iQ^TM^ SYBR Green Supermix (Bio-Rad, Hercules, CA) and normalized with respect to 16S rRNA whose levels did not vary under our experimental conditions [14].

### Protein overproduction and purification

The plasmid carrying the *‘lytM* gene (pAD05) was introduced into the BL21 λ DE3/pREP4 strain, allowing coproduction of the GroESL chaperonin in order to optimize recombinant protein solubility [66]. The resulting strains were grown in 2 liters of LB medium at room temperature, expression was induced during the mid-exponential growth phase by addition of 1 mM IPTG and incubation pursued for 4 hours. Protein purification by immobilized metal affinity chromatography (IMAC) was then performed as previously described [12]. Purified proteins were analyzed by SDS-PAGE on a 4–15% polyacrylamide gel (Bio-Rad, Hercules, CA).

### Western blotting experiments

Purified 'LytM was used to raise polyclonal rabbit antibodies, provided by Covalab (Villeurbanne, France) which were purified against 'LytM using the AminoLink® Kit from Thermo Scientific. Bacterial lysates were prepared from 45 ml cultures grown at 37°C with aeration in TSB + CdCl_2_ 0.25 µM +/− 1 mM IPTG until approximately OD_600 nm_ = 1. Cells were harvested by centrifugation (10 min, 5200×g), resuspended in 500 µl of 2X Laemmli SDS sample buffer and heated for 5 min at 99°C [67]. After centrifugation (10 min, 20,800×g), 20 µl of SDS-soluble proteins or purified ‘LytM (0.5 µg) were separated on a 12.5% SDS-PAGE gel, and transferred to a nitrocellulose membrane using a semi-dry blotter (Bio-Rad) and the following buffer: 25 mM Tris, 192 mM glycine, 20% ethanol. LytM, LytM H291A and purified ’LytM proteins were detected using the purified polyclonal rabbit antibodies (1:50,000), horseradish peroxidase-coupled anti-rabbit secondary antibodies (Zymed), and the Pico chemiluminescence Western blot kit (Pierce).

### Viability testing

Bacteria were stained using the LIVE/DEAD BacLight^TM^ viability assay L-7012 (Molecular Probes, Invitrogen, Carlsbad, CA). This stain distinguishes live cells from dead bacteria, based on membrane integrity and two nucleic acid stains. The green fluorochrome (SYTO 9) can penetrate intact membranes while the larger red fluorochrome (propidium iodide) only penetrates compromised membranes of dead bacteria. Bacterial cultures were grown in TSB CdCl2 (0.25 µM) +/− IPTG (1 mM). At appropriate optical densities, cultures were washed 3 times in 0.9% NaCl and concentrated in 0.9% NaCl between 2 to 10 times depending on the initial optical densities. Staining and fluorescent microscopic observations were then carried out as specified by the manufacturer.

### Electron microscopy

For ultrastructural analyses, bacteria were grown in TSB +/− 1 mM IPTG and 0.25 µM CdCl_2_ when indicated until OD_600_ = 1. Bacteria were fixed overnight at 4°C with 2.5% glutaraldehyde in 0.1 M cacodylate buffer, pH 7.4. Fixed samples were treated with 1% osmium tetroxide in 0.1 M cacodylate buffer, pH 7.4, for 1 h, dehydrated in ethanol and embedded in epoxy resin. Ultrathin sections were stained with 2% uranyl acetate and lead citrate and examined using a Jeol JEM1010 transmission electron microscope (Jeol, Tokyo, Japan) at 80 kV and an Eloise MegaView III camera (Eloise SARL, Roissy, France).

## Supporting Information

Table S1 **Oligonucleotides used in this study.**
(DOC)
