# Supplementary material for: Peptidoglycan Crosslinking Relaxation Plays an Important Role in Staphylococcus aureus WalKR-Dependent Cell Viability
Source: PLoS One. 2011 Feb 28;6(2):e17054. doi: 10.1371/journal.pone.0017054 (PMC3046168; doi:10.1371/journal.pone.0017054)
Supplement: Table S1 — Oligonucleotides used in this study. (DOC) [file pone.0017054.s001.doc]

**Supporting Table S1**. Oligonucleotides used in this study

| **Name** | Description | **Sequence*** |
| --- | --- | --- |
| **OAD007^a^** | *sceD* coding sequence | 5’-CCT*GGATCC*ATTTTATAGATGTTACAGTAG-3’ |
| **OAD008 ^a^** |  | 5’-CGA*GAATTC*CCCGATATATTCCGATCTA-3’ |
| **OAD023^a^** | *sle1* coding sequence | 5’-GGA*GGATCC*AATGTTAGGAAAGTTAAGCA-3’ |
| **OAD024^a^** |  | 5’-TAG*GAATTC*ATTATATATTTATATACG-3’ |
| **OAD028^a^** | *SAOUHSC_00773* coding sequence | 5’-A*GGATCC*ACAGCAAAGGAATGACAGCAAGATG-3’ |
| **OAD029 ^a^** |  | 5’-CCT*GAATT*CTAAACAGGCTAAGAGGATAGTCTTGTC-3’ |
| **OAD034 ^a^** | *SAOUHSC_02883* coding sequence | 5’-AA*GGATCC*GAGTCGCTAATGACTATATG-3’ |
| **OAD035 ^a^** |  | 5’-TC*GAATTC*GAGAATATTGACGTCATTTGATTTAC-3’ |
| **OAD042^a^** | *ssaA* coding sequence | 5’-TT*GGATCC*TGTCACACGGACATTACG-3’ |
| **OAD043^a^** |  | 5’-GA*GAATTC*GTTGGCGTAAAACATAGCCAT-3’ |
| **OAD054^a^** | *SAOUHSC_02576* coding sequence | 5’-TA*GGATCC*GATATAGAACTTTGGAGGACTT-3’ |
| **OAD055^a^** |  | 5’-AT*GAATTC*TGTACTAGAATTAAGTTTCAAGAC-3’ |
| **OAD056^a^** | *isaA* coding sequence | 5’-AC*GGATCC*CATAAACTTACAATTATTACTG-3’ |
| **OAD057^a^** |  | 5’-CCC*GAATTC*CTATGGGAAGAGCTATATATTTAATG-3’ |
| **OAD058^a^** | *SAOUHSC_00671* coding sequence | 5’-GTA*GGATCC*CATAATTTTAAGGAGGAGTCCCTTTG-3’ |
| **OAD059^a^** |  | 5’-CT*GAATTC*CTCACATGATGTGAATTGCTAGT-3’ |
| **OSA215^a^** | *lytM* coding sequence | 5’-AGT*GGATCC*GAAAACTAACTTTAACTTTAATGGAGGATG-3’ |
| **OSA217 ^a^** |  | 5’-CCA*GAATTC*GTATGATATTGTAACTTGGGATTTACTG-3’ |
| **OP274^a^** | *lss7* coding sequence | 5’-AGC*GGATCC*TTAGGAGGTATTAAGGTTG-3’ |
| **OSA252 ^a^** |  | 5’-A*GAATTC*TCACTTTATAGTTCCCCAAAGAACACC-3’ |
| **OSA259** | *lytM** mutagenesis | 5’-AGGCGCTGTTGAATTACCCGTACTACC-3’ |
| **OSA260** |  | 5’-CAACAGCGCCTGCCGTACACTTCCAACGTATG-3’ |
| **OSA265** | *ssaA** mutagenesis | 5’-CAAATACGTAGTATGTAGATTGACCAGAAGTG-3’ |
| **OSA266** |  | 5’-CATACTACGTATTTGATCGTGTAGGTGG-3’ |
| **OAD027^b^** | *‘lytM* coding sequence | 5’-G*CCATGG*CAGAAACGACAAACACCCAACA-3’ |
| **OAD018^b^** |  | 5’-CT*CTCGAG*TCTACTTTGCAAGTATGA-3’ |

| **OAD003^c^** | *lytM* upstream region | 5’-GCA*GGATCC*GCTCAAAACAATGATAAAGAAATGG-3’ |
| --- | --- | --- |
| **OAD004 ^c^** |  | 5’-TAT*CCATGG*TCCTCCATTAAAGTTAAAGTTAG-3’ |
| **OAD013 ^c^** | *lytM* downstream region | 5’-CG*CCATGG*TTGCAAAGTAGATAATACAGTAAATCCC-3’ |
| **OAD014 ^c^** |  | 5’-ATG*AGATCT*ATATATGCACTTGCTATGGGTATCG-3’ |
| **OAD064^c^** | *ssaA* upstream region | 5’-AA*GGATCC*GATTTACTCCTGTTTGTCTTTCTC-3’ |
| **OAD065^c^** |  | 5’-CTA*GTCGAC*TTTCGTAATGTCCGTGTGACAA-3’ |
| **OAD066^c^** | *ssaA* downstream region | 5’-CAT*GTCGAC*ATTAGTGATGGCTATGTTTTACGC-3’ |
| **OAD067^c^** |  | 5’-TA*CCATGG*GGAACATCAGGTATTTACTATACG-3’ |
| **OSA119** | *ssaA* intragenic region (qRT-PCR) | 5’-CCGTACTGGTGGTTTAGGTGCAAGCTACAG-3’ |
| **OSA120** |  | 5’-GCATTGCCCCAAGTTGAACCGATTTTACCA-3’ |
| **OSA158** | *walK* intragenic region (qRT-PCR) | 5’-TACAATCCCTTCATACTAAACTTGTAATTG-3’ |
| **OSA159** |  | 5’-GTGCATTTACGGAGCCCTTTTCGTCATATA-3’ |
| **OSA161** | 16S rRNA intragenic region (qRT-PCR) | 5’-ACGTGGATAACCTACCTATAAGACTGGGA-3’T |
| **OSA162** |  | 5’-TACCTTACCAACTAGCTAATGCAGCG-3’ |
| **OSA218** | *lytM* intragenic region (qRT-PCR) | 5’-AGCGAACAGTAATAACTACCAATG-3’ |
| **OSA219** |  | 5’-CGATGCCACCAGACATACG-3’ |

*Added restriction site sequences are shown in italics

^a^ Oligonucleotides for gene amplification and CdCl_2_-dependent expression in *S*. *aureus*.

^b^ Oligonucleotides for *lytM* amplification and insertion into pET28/16 for protein overexpression and purification.

^c^ Oligonucleotides for *lytM/ssaA* deletion.
